# A Meta-Analysis of Oxidative Stress Markers in Depression

**DOI:** 10.1371/journal.pone.0138904

**Published:** 2015-10-07

**Authors:** Tao Liu, Shuming Zhong, Xiaoxiao Liao, Jian Chen, Tingting He, Shunkai Lai, Yanbin Jia

**Affiliations:** 1 Department of Psychiatry, First Affiliated Hospital, Jinan University, Guangzhou, China; 2 Guangzhou Brain Hospital, Guangzhou, China; 3 First School of Clinical Medicine, Jinan University, Guangzhou, China; 4 Management School, Jinan University, Guangzhou, China; Baylor College of Medicine, UNITED STATES

## Abstract

**Object:**

Studies have suggested that depression was accompanied by oxidative stress dysregulation, including abnormal total antioxidant capacity (TAC), antioxidants, free radicals, oxidative damage and autoimmune response products. This meta-analysis aims to analyse the clinical data quantitatively by comparing the oxidative stress markers between depressed patients and healthy controls.

**Methods:**

A search was conducted to collect the studies that measured the oxidative stress markers in depressed patients. Studies were searched in Embase, Medline, PsychINFO, Science direct, CBMDisc, CNKI and VIP from 1990 to May 2015. Data were subjected to meta-analysis by using a random effects model for examining the effect sizes of the results. Bias assessments, heterogeneity assessments and sensitivity analyses were also conducted.

**Results:**

115 articles met the inclusion criteria. Lower TAC was noted in acute episodes (AEs) of depressed patients (*p*<0.05). Antioxidants, including serum paraoxonase, uric acid, albumin, high-density lipoprotein cholesterol and zinc levels were lower than controls (*p*<0.05); the serum uric acid, albumin and vitamin C levels were increased after antidepressant therapy (*p*<0.05). Oxidative damage products, including red blood cell (RBC) malondialdehyde (MDA), serum MDA and 8-F_2_-isoprostanes levels were higher than controls (*p*<0.05). After antidepressant medication, RBC and serum MDA levels were decreased (*p*<0.05). Moreover, serum peroxide in free radicals levels were higher than controls (*p*<0.05). There were no differences between the depressed patients and controls for other oxidative stress markers.

**Conclusion:**

This meta-analysis supports the facts that the serum TAC, paraoxonase and antioxidant levels are lower, and the serum free radical and oxidative damage product levels are higher than controls in depressed patients. Meanwhile, the antioxidant levels are increased and the oxidative damage product levels are decreased after antidepressant medication. The pathophysiological relationships between oxidative stress and depression, and the potential benefits of antioxidant supplementation deserve further research.

## Introduction

Depression affects millions of people and is the leading global cause of disability according to the World Health Organization [1]. However, the psychopathological mechanisms of depression are unclear. Recently, many studies have indicated that oxidative stress might play a vital role [2]. Some studies have demonstrated that depressed patients’ oxidative product levels in their peripheral blood [3, 4], red blood cells (RBC) [4], mononuclear cells [5], urine [6], cerebrospinal fluid [7] and postmortem brains [8] were abnormal. Antioxidant system disturbance in peripheral blood has also been reported [9]. Autoimmune responses against neoepitopes induced by oxidative damage of fatty acid and protein membranes have been reported [10, 11]. Lower glutathione (GSH) levels [12] and a negative relationship between anhedonia severity and occipital GSH levels [13] were found through magnetic resonance spectroscopy (MRS).

Oxidative stress is defined as a persistent imbalance between oxidation and anti-oxidation, which leads to the damage of cellular macromolecules [14, 15]. The free radicals consist of reactive oxygen species (ROS) and reactive nitrogen species (RNS). The main ROS includes superoxide anion, hydroxy radical and hydrogen peroxide, and the RNS consists of nitric oxide (NO), nitrogen dioxide and peroxynitrite. Nitrite is often used as a marker of NO activity. Interestingly, the brain appears to be more susceptible to the ROS/RNS because of the high content of unsaturated fatty acids, high oxygen consumption per unit weight, high content of key ingredients of lipid peroxidation (LP) and scarcity of antioxidant defence systems [16]. The oxidative products include products of oxidative damage of LP, protein and DNA in depression. As a product of LP, abnormal malondialdehyde (MDA) levels in depression have been reported [17]. 8-F_2_-isoprostane (8-*iso*-PGF_2α_) is another product of LP [18] that is considered to be a marker of LP because of its chemical stability [19]. The protein carbonyl (PC), 8-hydroxy-2-deoxyguanosine (8-OHdG) and 8-oxo-7, 8-dihydroguanosine (8-oxoGuo) are the markers of protein, DNA and RNA oxidative damage, respectively [3, 20, 21]. The oxidative damage to cellular macromolecules changes the structure of original epitopes, which leads to the generation of new epitopes modified (neoepitopes). The antibodies against oxidative neoepitopes in depression have been found [10, 11, 22–24]. On the other hand, the antioxidant defence systems can be divided into enzymatic and non-enzymatic antioxidants. The non-enzymatic antioxidants include vitamins C and E, albumin, uric acid, high-density lipoprotein cholesterol (HDL-C), GSH, coenzyme Q10 (CoQ10), ceruloplasmin, zinc, selenium, and so on. The enzymatic antioxidants include superoxide dismutase (SOD), glutathione peroxidase (GPX), catalase (CAT), glutathione reductase (GR), paraoxonase 1 (PON1), and so on.

Some studies have reported that patients of depression have significant alterations in total antioxidant capacity (TAC), antioxidants, free radicals, oxidative products and antibodies against oxidative neoepitopes, but these findings were not consistent. A previous quantitative review of the association between depression and oxidative stress markers was reported, but it comprehensively analysed all oxidative stress markers at once [25]. The objective of this study is to review the studies of each oxidative stress marker in depression and to quantify the magnitude of differences in oxidative stress markers between the depressed patients and control subjects in different sample sources (e.g., serum, plasma, RBCs) in acute episodes (AEs). Considering the effects of the treatment settings, we quantified the changes after antidepressant therapy. No differences in oxidative stress markers between serum and plasma were found in our analyses, both materials were referred to as “serum”.

## Methods and Materials

### Search procedures

We searched Medline, Embase, PsychINFO, Sciencedirect, CBMDisc, CNKI and VIP from 1990 to May 2015 using the following key words: (depression OR major depression OR unipolar depression OR major depressed disorder) AND (oxidation OR oxidative stress OR antioxidant OR antioxidant enzyme OR total antioxidant capacity OR total antioxidant potential OR free radical OR superoxide dismutase OR glutathione peroxidase OR catalase OR paraoxonase OR glutathione reductase OR vitamin C OR vitamin E OR albumin OR uric acid OR high-density lipoprotein cholesterol OR zinc OR nitric oxide OR nitrite OR peroxide OR malondialdehyde OR 8-F_2_-isoprostane OR protein carbonyl). We reviewed the titles and abstracts to select potentially relevant papers. If there was doubt about the suitability of the paper based on the abstract, the full text was reviewed. We manually searched the references and relevant articles for inclusion.

### Inclusion criteria

Studies were included in our analyses if they met the following criteria: 1) cross-sectional studies measuring oxidative stress markers in serum, plasma, or RBC of depressed patients; 2) studies that assessed oxidative stress markers in patients with an acute exacerbation of depression at baseline and again after antidepressant therapy; 3) inclusion of a depressed group as diagnosed by standard recognised criteria or screened with a standardized instrument; or 4) studies that provided subject numbers, means and standard deviations. Through our search strategy, we decided to focus on TAC, certain enzymatic antioxidants (SOD, GPX, CAT, PON and GR), non-enzymatic antioxidants (albumin, uric acid, zinc, HDL-C, vitamin C and E), free radicals (NO, nitric oxide, peroxide) and oxidative damage products (MDA, 8-*iso*-PGF_2α_, PCC), but not antibodies against oxidative neoepitopes and other oxidative stress markers because of limited studies.

### Exclusion criteria

Studies were excluded from our analyses if they met the following criteria: 1) reviews, conference abstracts, editorials and letters; 2) animal studies; 3) studies that reported on depressed symptoms in the context of other neuropsychiatric disorders or medical illnesses; or 4) dual publications (if the same sample was used in more than one publication, the study that provided stronger evidence was considered for analysis).

### Data extraction and quality assessment

The data were extracted by two independent raters (TL and SMZ), with disagreements settled by discussion. Information was extracted in a systematic way as follows: 1) population characteristics; 2) sample types; 3) data for mean (SD); 4) diagnostic strategy; 5) treatment situations and 6) confounding factors. The quality was assessed independently by two raters by using the Newcastle-Ottawa Scale (NOS) (case control studies or cohort studies) [26]. The NOS of case control studies assesses three components: selection, comparability and exposures, and the NOS of cohort studies assesses three components: selection, comparability and outcomes. We identified “high”-quality with a “star”. A study could be awarded a maximum of one star for each numbered item within the “selection” and “exposure or outcomes” categories. A maximum of two stars could be given for “comparability”. Studies with ≤ 4 stars were considered low quality and were excluded.

### Statistical analysis

All statistical analyses were performed using the standardized mean difference (SMD) methodology in the Stata 12.0 software (StataCorp, College Station, Texas). Pooled effect sizes (ES) were calculated according to DerSimonian and Laird for the random effects model because of the diversity of methods, patients’ clinical statuses and treatments [27]. Potential publication bias was assessed by using Egger’s test [28]. Between-study heterogeneity (I^2^) was assessed as previously described by Glasziou and Sanders [29]. Sensitivity analysis was performed by removing each study one by one and all combinations of two studies. The significance was defined as *p*<0.1 in Egger’s test and the significance of the other statistical tests was defined *p*<0.05. All comparisons were two-tailed, and 95% confidence intervals (CI) were described where applicable.

## Results

### Systematic review

The search identified 7443 potentially relevant articles. After we removed duplicates, 6038 articles were remained. On the initial screening, 5894 were excluded based on titles and abstracts. Full-text evaluation was conducted for the remaining 144 articles, and 25 articles were excluded for not fulfilling inclusion criteria. 119 articles were remained. However, the quality of four papers was low, ranging from 3 to 4 stars in total [30–33]. Eventually, 115 articles that included 273 studies were included to our analyses [4, 9, 11, 17, 18, 20, 34–142]. 22 comparison analyses of depressed patients and healthy controls, and 14 comparison analyses of pre-and post-therapy in depressed patients were performed. Table 1 presents characteristics of included studies and meta-analyses of oxidative stress markers.

**Table 1:** Meta-Analyses of Oxidative Stress Marker Levels.

Oxidative			N	Post-Treatment					Egger's Test		Heterogeneity Heterogeneity			
Stress Markers	Samples	Clinical Status	studies	Patients	Patients	Controls	Mean ES (95% CI)	*p* Value	t	*p* Value	χ2	*p* Value	I^2^ (%)	References
**TAS**	Serum	Acute Episodes	11	674		591	-0.538 (-1.001, -0.075)	0.023	0.01	0.989	135.21	0.000	92.6	9, 34–42
		Treatment	7	285	261		0.069 (-0.306, 0.444)	0.719	1.42	0.215	27.70	0.000	78.3	9, 34, 35, 40, 41, 43
**Vitamin C**	Serum	Acute Episodes	7	357		276	-0.501 (-1.323, 0.321)	0.232	-0.55	0.603	133.28	0.000	95.5	9, 34, 36, 39, 52, 53, 58
		Treatment	4	132	180		1.473 (0.177, 2.769)	0.026	-4.38	0.048	56.20	0.000	94.7	9, 34, 52, 58
**Vitamin E**	Serum	Acute Episodes	6	274		214	0.265 (-0.884, 1.414)	0.651	-3.58	0.023	185.00	0.000	97.3	9, 17, 34, 36, 58, 59
		Treatment	3	128	104		0.237 (-0.556, 0.082)	0.146	-0.53	0.687	4.02	0.134	50.2	9, 34, 58, 59
**Uric Acid**	Serum	Acute Episodes	12	762		517	-0.695 (-1.242, -0.149)	0.013	-4.23	0.002	236.06	0.000	95.3	34, 36, 39, 60–66
		Treatment	4	209	185		3.721 (1.756, 5.687)	0.000	2.58	0.123	107.45	0.000	97.2	34, 60, 62
**Albumin**	Serum	Acute Episodes	27	983		712	-0.820 (-1.135, -0.505)	0.000	-2.83	0.009	239.59	0.000	89.1	34, 36, 39, 51, 56, 59, 67–80
		Treatment	7	260	193		0.667 (0.025, 1.325)	0.042	-1.12	0.312	56.28	0.000	89.3	34, 74, 78, 80
**HDL-C**	Serum	Acute Episodes	46	2914		4475	-0.360 (-0.560, -0.159)	0.000	-1.96	0.057	576.20	0.000	92.2	34, 51, 56, 57, 59, 76, 80–114
		Treatment	4	174	174		-0.109 (-0.405, 0.187)	0.469	-1.92	0.306	4.79	0.187	37.4	56, 80, 85, 115
**Zinc**	Serum	Acute Episodes	21	1119		725	-1.037 (-1.348, -0.725)	0.000	-0.54	0.599	117.71	0.000	83.9	59, 70–72, 79, 116–129
**SOD**	RBC	Acute Episodes	9	219		284	0.096 (-0.635, 0.828)	0.797	-0.58	0.583	97.94	0.000	91.8	4, 9, 34, 35, 44–47
		Treatment	7	245	221		-0.195 (-0.609, 0.219)	0.357	-0.16	0.175	26.53	0.000	77.4	9, 34, 35, 43–45
**GPX**	RBC	Acute Episodes	5	145		123	-0.518 (-1.597, 0.560)	0.346	-1.37	0.265	64.47	0.000	93.8	4, 34, 35, 44
		Treatment	4	182	158		-0.052 (-0.306, 0.202)	0.687	-3.62	0.069	4.10	0.251	26.8	34, 35, 43, 44
**CAT**	RBC	Acute Episodes	4	88		68	0.182 (-0.483, 0.847)	0.591	-1.28	0.329	12.66	0.005	76.3	35, 44, 45
		Treatment	4	140	140		0.051 (-0.183, 0.286)	0.667	18.82	0.003	0.05	0.997	0.0	35, 43–45
**GR**	RBC	Acute Episodes	3	45		49	2.374 (-0.275, 4.968)	0.079	10.44	0.061	56.25	0.000	96.4	4, 44
**SOD**	Serum	Acute Episodes	8	295		234	1.021 (-0.063, 2.105)	0.065	0.64	0.548	187.96	0.000	96.3	45, 48–54
		Treatment	4	90	82		-0.446 (-1.496, 0.603)	0.405	1.06	0.401	8.91	0.000	89.6	45, 49, 50, 52
**GPX**	Serum	Acute Episodes	7	270		305	-0.394 (-0.959, 0.171)	0.172	-0.47	0.659	56.28	0.000	89.3	36, 38, 44, 47, 53, 55
**CAT**	Serum	Acute Episodes	2	52		56	0.876 (-1.375, 3.126)	0.446			28.18	0.000	96.5	48, 51
**PON**	Serum	Acute Episodes	3	165		265	-0.263 (-0.463, -0.063)	0.010	-0.75	0.989	0.66	0.718	0.0	34, 56, 57
		Treatment	2	74	67		-0.035 (-0.296, 0.366)	0.837			0.37	0.540	0.0	34, 56
**Nitrite**	Serum	Acute Episodes	10	390		425	-0.325 (-0.883, 0.233)	0.254	-1.52	0.166	130.24	0.000	93.1	49, 57, 130–135
		Treatment	2	49	49		-0.650 (-1.057, -0.243)	0.002			0.19	0.659	0.0	49, 133
**NO**	Serum	Acute Episodes	7	299		270	-0.607 (-2.276, 1.062)	0.476	-0.43	0.686	346.47	0.000	98.3	9, 53, 130, 136–139
**Peroxide**	Serum	Acute Episodes	4	203		108	1.525 (0.472, 2.577)	0.005	4.93	0.039	38.46	0.000	92.2	4, 11, 24, 39
**MDA**	RBC	Acute Episodes	3	95		77	2.379 (0.816, 3.942)	0.003	70.64	0.009	29.38	0.000	93.2	4, 35, 44
		Treatment	4	157	157		0.934 (0.700, 1.167)	0.000	0.56	0.630	0.64	0.887	0.0	35, 43, 44
**MDA**	Serum	Acute Episodes	10	498		498	0.993 (0.378, 1.607)	0.002	3.68	0.006	178.71	0.000	95.0	9, 17, 34, 37, 44, 52–54, 140
		Treatment	4	142	118		1.787 (0.048, 3.526)	0.044	2.69	0.115	123.33	0.000	97.6	9, 34, 44, 52
**8-*iso*-PGF**_**2α**_	Serum	Acute Episodes	3	125		124	0.622 (0.054, 1.190)	0.032	-0.40	0.756	8.12	0.017	75.4	18, 141, 142
**PCC**	Serum	Acute Episodes	5	307		416	0.477 (-0.309, 1.263)	0.234	-0.16	0.900	97.97	0.000	97.5	20, 37, 51, 61

N, number;

ES, effect sizes;

CI, confidence interval;

AEs, acute episodes;

RBC, red blood cell;

TAC, total antioxidant capacity;

SOD, superoxide dismutase;

GPX, glutathione peroxidase;

CAT, catalase;

GR, glutathione reductase;

PON, paraoxonase;

HDL-C, high-density lipoprotein cholesterol;

NO, nitric oxide;

MDA, malondialdehyde;

8-*iso*-PGF_2α,_ 8-F_2_-isoprostanes;

PCC, oxidation protein product.

### Studies of TAC

**Fig 1:**
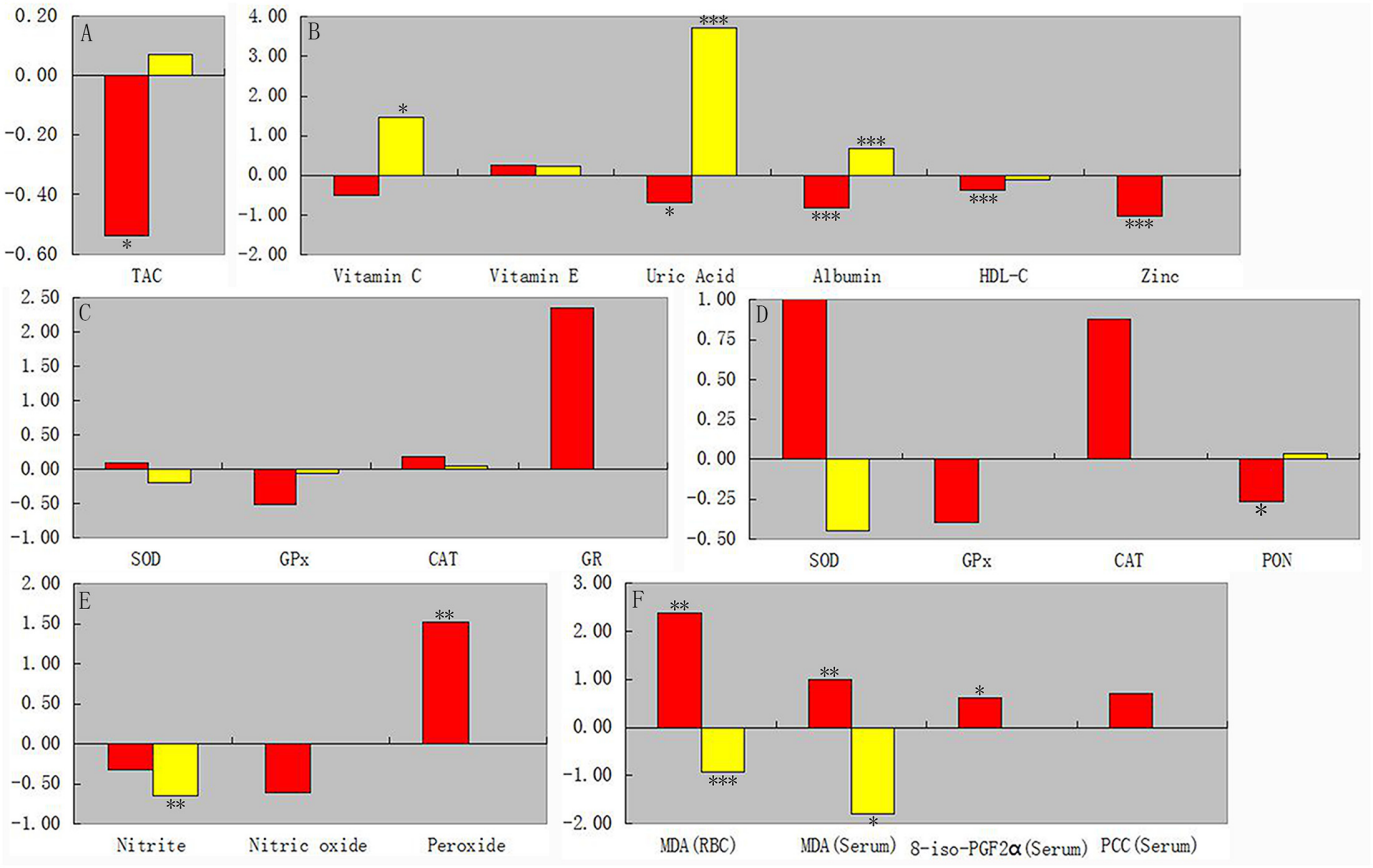
The comparisons of effect sizes for serum TAC (A), serum non-enzymatic antioxidants (B), red blood cell enzymatic antioxidants (C), serum enzymatic antioxidants (D), serum free radicals (E) and serum oxidative damage products (F) in acute episodes between depressed patients and controls (red bar) and in comparison before and after treatment of depressed patients (yellow bar). Positive effect sizes (bars go upwards) indicate that the marker levels in depressed patients were higher than controls or increased after antidepressant therapy; negative effect sizes (bars go downwards) indicate that marker levels were lower than controls or decreased after antidepressant therapy. *p < 0.05, **p < 0.01, ***p < 0.001. TAC, total antioxidant capacity; HDL-C, high-density lipoprotein cholesterol; SOD, superoxide dismutase; GPX, glutathione peroxidase; CAT, catalase; GR, glutathione reductase; PON, paraoxonase; MDA, malondialdehyde; 8-*iso*-PGF_2α_, 8-F2-isoprostanes; PCC, oxidation protein product.

The serum TAC was lower in AEs of depressed patients than controls (*p*<0.05), but it did not increase after antidepressant therapy (Fig 1A). Publication bias assessed with Egger’s test was not significant for all analyses. The heterogeneity was high in the ES estimates for all analyses. Sensitivity analysis of serum TAC in the comparison of before and after the treatment (CBAT) was performed by removing two studies [34, 40], the heterogeneity was no longer significant, and the result remained unchanged. The heterogeneity was also significant for analysis of serum TAC in AEs after removing each study one by one and all combinations of two studies.

### Studies of non-enzymatic antioxidants

The serum uric acid, albumin, HDL-C and zinc levels were lower in AEs than controls (*p*<0.05), and uric acid, albumin and vitamin C levels were increased after antidepressant therapy (*p*<0.05). There were no differences in vitamin C and E levels between groups (Fig 1B). Publication bias was significant for vitamin C in CBAT, vitamin E in AEs, uric acid in AEs, albumin in AEs and CBAT, and HDL-C in AEs analyses (*p*<0.1), but not for other analyses. Heterogeneity was low for HDL-C in CBAT and moderate for vitamin C in CBAT but high in the other analyses. Sensitivity analyses showed heterogeneity was no longer significant and that vitamin C levels remained increased after antidepressant therapy after two studies were removed [34, 58] (*p*<0.001). There was also significant heterogeneity for other analyses that could be performed after removing each study one by one and all combinations of two studies.

### Studies of enzymatic antioxidants

#### RBC enzymatic antioxidants

There were no differences in all RBC enzymatic antioxidant activities in all analyses (Fig 1C). Publication bias was significant for GPX in CBAT, CAT in CBAT and GR in AEs (*p*<0.1), but not in other analyses. Heterogeneity was high for all enzymatic antioxidant analyses except CAT in CBAT. Sensitivity analyses showed that heterogeneity was no longer significant, SOD activity was decreased after antidepressant therapy after two studies were removed [43, 45] (*p*<0.05), and GPX activity was higher than controls after two studies were removed [4, 34] (*p*<0.05), CAT activity still didn’t differ between groups after one study was removed [35]. There was also significant heterogeneity for other analyses after each single study and all combinations of two studies were removed.

#### Serum enzymatic antioxidants

Serum PON activity was lower in AEs than controls, but it didn’t increased after antidepressant therapy. There were no differences in the other serum enzymatic antioxidant activities (Fig 1D). Publication bias was not significant for all enzymatic antioxidant analyses that could be performed. Heterogeneity was high for all analyses except PON activity in AEs and CBAT. Sensitivity analyses showed that heterogeneity was no longer significant, SOD activity was decreased after antidepressant therapy after one study was removed [49] (*p*<0.01). There was also significant heterogeneity for other analyses that could be performed after removing each study one by one and all combinations of two studies.

### Studies of free radicals

The serum peroxide levels were higher in AEs than controls (*p*<0.05). Serum nitrite levels were decreased after antidepressant therapy (*p*<0.01). There were no differences in serum nitrite and NO levels between groups (Fig 1E). Publication bias was significant for peroxide levels in AEs (*p*<0.1), but not in any of the other analyses that could be performed. The heterogeneity was significant for nitrite, NO and peroxide levels in AEs but not for nitrite levels in CBAT. Sensitivity analyses showed that heterogeneity was no longer significant, the peroxide levels in AEs were still higher after one study was removed [4] (*p*<0.01). There was also significant heterogeneity in other analyses after removing each study one by one and all combinations of two studies.

### Studies of oxidative damage products

The RBC MDA, serum MDA and 8-*iso*-PGF_2α_ levels in AEs were all higher than controls (*p*<0.05), the RBC and serum MDA levels were decreased after antidepressant therapy (*p*<0.05). The serum PCC levels didn’t differ between groups in AEs (Fig 1F). Publication bias was significant for RBC and serum MDA in AEs (*p*<0.1), but not for other analyses. There was significant heterogeneity for all oxidative damage product analyses except the RBC MDA in CBAT. Sensitivity analyses showed that heterogeneity was no longer significant, RBC MDA levels in AEs was still higher than controls after one study was removed [4] (*p*<0.01), serum MDA levels remained decreased after antidepressant therapy after two studies were removed [4, 52] (*p*<0.01). Serum PCC levels in AEs still didn’t differ between groups after one study was removed [20]. In other sensitivity analyses, the heterogeneity was also significant.

## Discussion

The present findings support oxidative stress may be disordered in depressed patients, which is demonstrated by abnormal oxidative stress marker levels. In this meta-analysis, at first we found in depressed patients: 1) the serum TAC, PON, uric acid, albumin, HDL-C and zinc levels were lower than controls; 2) the serum peroxide, MDA, 8*-iso*-PGF_2α_ and RBC MDA levels were higher than controls. To explore the effect of the antidepressant therapy to oxidative stress markers, we reviewed the studies which had changes. And it came to the conclusions: 1) the serum uric acid, albumin, and vitamin C levels were increased; 2) the serum nitrite, RBC and serum MDA levels were decreased.

Considering the between-study heterogeneity, we performed sensitivity analyses. Following the sensitivity analyses, the heterogeneity of some oxidative markers was significantly decreased, the RBC GPX activities changed to be higher than controls, and the RBC and serum SOD activities were decreased after antidepressant therapy. However, the heterogeneity of most oxidative markers was also obvious, and even though the heterogeneity of a few studies was significantly decreased, but the results remained unchanged after one or two studies were removed. The higher RBC GPX activity could have been a compensatory mechanism for the excess production of free radicals in depressed patients [35]. The decreased RBC and serum SOD activities could reflect a decrease in oxidative stress disturbance. PON1 is an enzymatic antioxidant that bound to HDL. Most of antioxidant activity of HDL relies on PON1 to prevent LDL and HDL oxidation [143]. Lower PON1 activity in depressed patients may be ascribed to lower HDL-C levels.

The limitations of the present study include minor studies, publication bias and between-study heterogeneity in some analyses; thus, the results for many oxidative stress markers should be interpreted with caution. Because of the effect of the sample sources on oxidative stress markers, we quantified the magnitude of differences in different sample sources. However, there were few published oxidative stress marker studies, the number of included studies was small. Twenty-two comparison analyses of the depressed patients and healthy controls in oxidative stress markers were performed in this study, but one, four and two of the 22 comparison analyses only included 2, 3, and 4 studies, respectively. Fourteen analyses compared the effect of antidepressant therapy in depressed patients, and two, two and seven of this 14 comparison analyses only included 2, 3, and 4 studies, respectively.

It is possible that the heterogeneity could also have been attributable to many factors, including unmatched age, gender, race, ethnicity, body mass index (BMI), smoking, dietary habits, treatment settings, different assay methodologies, different phases of illness and different clinical courses of illness [144]. By quality assessment, we eliminated a number of low-quality studies. However, most studies considered some, but not all confounding factors so that we could not exclude all of these studies. For example, 10 articles that measured serum TAC in AEs in our analyses, 9 of them considered the effects of age and gender [9, 34–36, 38–42], and only 2 articles matched BMI and smoking status [34, 39]. There are data that link oxidative stress and nicotine dependence [138, 145], but a sub-analysis was not possible for the limited number of studies that stratified by smoking status. Moreover, treatment settings factor was complex. Because most studies included patients that treated with various agents and treatment durations, the effects of specific antidepressant agents and the duration of antidepressant therapy on individual oxidative stress markers could not be evaluated exactly.

The serum antioxidant levels are significantly lower in depression in our study and previous reports, including PON, albumin, zinc, uric acid HDL-C, CoQ10 [146] and GSH [4, 38]. Meanwhile, the oxidative damage product levels are significantly higher. The body couldn’t scavenge the excess free radicals (peroxide), leading to damages of main parts of cellular macromolecules such as fatty acids, protein, DNA, RNA and mitochondria. The longitudinal antidepressant therapy can reverse these abnormal oxidative stress parameters. It has proved these phenomena occur in depression, such as increased levels of MDA, 8-*iso*-PGF_2α_, 8-oxoGuo and 8-OHdG [3, 21]. Oxidative stress plays a crucial role in the pathophysiology of depression. Some genes may be a potential factor. Lawlor et al (2007) reported the R allele of PON1Q192R was associated with depression [147]. In addition, poor appetite, psychological stressors, obesity, metabolic syndrome, sleep disorders, cigarette smoking and unhealthy lifestyle may also contribute to it [148]. Furthermore, oxidative stress activates the immune-inflammatory pathways [148]. But some studies supported decrease in albumin, zinc and HDL-C levels was probably related to increased levels of pro-inflammatory cytokines (such as interleukin-1 (IL-1) and IL-6) [59, 70–72, 117] during an acute phase response, which illustrated the activated immune-inflammatory pathways also activates the oxidative stress. These two mechanisms influence each other. On the other hand, the oxidative damage to cellular macromolecules changes the structure of original epitopes, which leads to generation of new epitopes modified (neoepitopes). Oxidative neoepitopes reported up to now include the conjugated oleic and azelaic acid, MDA, phosphatidyl inositol (Pi), NO-modified adducts and oxidized low density lipoprotein (oxLDL) [11, 22–24]. Maes et al reported the levels of serum IgG antibody against the oxLDL and IgM antibodies against the conjugated oleic and azelaic acid, MDA, Pi and NO-modified adducts were increased in depression [11, 22–24]. Depleted antioxidant defence in depression suggests that antioxidant supplements may be useful in clinical management. Preliminary evidence has indicated that patients treated with CoQ10 showed improvement in depressive symptoms and decrease in hippocampal oxidative DNA damage [149]. In our analyses, vitamin C and E levels did not differ between depressed patients and controls, but many studies have reported that vitamin C and E supplements could improve depressive moods [150, 151].

## Conclusion

In conclusion, we should cautiously interpret these results because the limitations of minor studies, public bias and between-study heterogeneity in our study. The results of our meta-analysis reveal that oxidative stress is disturbed in patients of depression. Further investigation needs to explore the roles of oxidative stress markers in the pathophysiology of depression and the potential benefits of antioxidant supplementation.

## Supporting Information

S1 PRISMA Checklist Prisma 2009 checklist. (DOC)
