# Supplementary material for: A Meta-Analysis of Oxidative Stress Markers in Depression
Source: PLoS One. 2015 Oct 7;10(10):e0138904. doi: 10.1371/journal.pone.0138904 (PMC4596519; doi:10.1371/journal.pone.0138904)
Supplement: S1 PRISMA Checklist — (DOC) [file pone.0138904.s001.doc]

| **Section/topic** | **#** | **Checklist item** | **Reported on page #** |
| --- | --- | --- | --- |
| **TITLE** | | |  |
| Title | 1 | A Meta-Analysis of Oxidative Stress Markers in Depression | 1 |
| **ABSTRACT** | | |  |
| Structured summary | 2 | ***Object:*** Studies have suggested that depression was accompanied by oxidative stress dysregulation, including abnormal total antioxidant capacity (TAC), antioxidants, free radicals, oxidative damage and autoimmune response products. This meta-analysis aims to quantitatively analyse the clinical data by comparing the oxidative stress markers in peripheral blood between depressive patients and healthy controls.  ***Methods:*** A search was conducted to identify those studies that measured the oxidative stress markers in depressed patients. Studies were searched for in Embase, Medline, PsychINFO, Science direct, CBMDisc, CNKI and VIP from 1990 to May 2015. Data were subjected to meta-analysis by using a random-effects model to examine the effect sizes of the poor results. Bias assessments, heterogeneity assessments (I2) and sensitivity analyses were conducted.  ***Results:*** We obtained 125 articles that met inclusion criteria. Lower TAC was noted in acute episodes (AEs) of depressed patients (p<0.05). Antioxidants, including serum paraoxonase activity, uric acid, albumin, high-density lipoprotein cholesterol and zinc levels were lower in depressive patients than controls (p<0.05 for each); the serum uric acid, albumin and vitamin C levels were increased after antidepressant therapy (p<0.001 for each). Oxidative damage products, including red blood cell (RBC) malondialdehyde (MDA), serum MDA and 8-F2-isoprostanes levels were higher than controls (p<0.05 for each). After antidepressant medication, RBC and serum MDA levels were decreased (p<0.05 for each). Moreover, serum peroxide in free radicals levels were higher than controls (p<0.05). There were no differences between depressed patients and controls for other oxidative stress markers.  ***Conclusion:*** This meta-analysis supports the fact that the serum TAC, paraoxonase activity and antioxidant levels are lower, and the serum free radical and oxidative damage product levels are higher than controls. Moreover, the antioxidant levels are increased and the oxidative damage product levels are decreased after antidepressant medication. The pathophysiological relationships between oxidative stress and depression, and the potential benefits of antioxidant supplementation deserve further research. | 1 |
| **INTRODUCTION** | | |  |
| Rationale | 3 | Depression affects millions of people and is the leading global cause of disability according to the World Health Organization [1]. However, the psychopathological mechanisms of depression are unclear. Recently, many studies have indicated that oxidative stress might play a vital role [2]. Some studies have demonstrated that depressed patients’ oxidative product levels in their peripheral blood [3, 4], red blood cells (RBC) [4], mononuclear cells [5], urine [6], cerebrospinal fluid [7] and postmortem brains [8] were abnormal. Antioxidant system disturbance in peripheral blood has also been reported [9]. Autoimmune responses against neoepitopes induced by oxidative damage from fatty acid and protein membranes have been reported [10, 11]. Lower glutathione (GSH) levels [12] and a negative relationship between anhedonia severity and occipital GSH levels [13] were found by magnetic resonance spectroscopy.  Oxidative stress is defined as a persistent imbalance between oxidation and anti-oxidation when antioxidant defence systems can’t remove excess free radicals, including the reactive oxygen species (ROS) and reactive nitrogen species (RNS), which leads to the damage of cellular macromolecules [14, 15]. Interestingly, the brain appears to be more susceptible than other organs to the ROS/RNS because of the high content of unsaturated fatty acids, high oxygen consumption per unit weight, high content of key ingredients of lipid peroxidation (LP) and scarcity of antioxidant defence systems [16]. The ROS includes superoxide anion, hydroxy radical and hydrogen peroxide, and the RNS consists of nitric oxide (NO), nitrogen dioxide and peroxynitrite. Nitrite is often used as a marker of NO activity. The oxidative products that have been reported were products of LP, protein, DNA and RNA oxidative damage in depression. As a product of LP, abnormal malondialdehyde (MDA) levels in depression have been reported [17]. 8-F2-isoprostane (8-iso-PGF2α) is also a product of LP [18] that is considered to be a quantitative marker of LP because of its chemical stability [19]. The protein carbonyl (PC), 8-hydroxy-2-deoxyguanosine (8-OHdG) and 8-oxo-7, 8-dihydroguanosine (8-oxoGuo) are the markers of protein, DNA and RNA oxidative damage, respectively [3, 20, 21]. New epitopes (neoepitopes) modified are generated because oxidative damage to cellular macromolecules changes structure of original epitopes. The antibodies to oxidative neoepitopes in depression have been reported [10, 11, 22-24]. The antioxidant defence systems can be divided into enzymatic and non-enzymatic antioxidants. The major cellular non-enzymatic antioxidants are vitamins C and E, albumin, uric acid, high-density lipoprotein cholesterol (HDL-C), GSH, coenzyme Q10 (CoQ10), ceruloplasmin, zinc and selenium, and the enzymatic antioxidants include superoxide dismutase (SOD), glutathione peroxidase (GPX), catalase (CAT), glutathione reductase (GR) and paraoxonase 1 (PON1). | 2 |
| Objectives | 4 | Some studies have reported that patients with depression have significant alterations in total antioxidant capacity (TAC), antioxidants, free radicals, oxidative products and antibodies to oxidative neoepitopes, but these findings were not consistent. A previous systematic, quantitative review of the association between depression and oxidative stress markers was reported, but it comprehensively analysed all oxidative stress markers at once [25]. The objective of this study, therefore, is to review the studies of oxidative stress marker in depression and to quantify the magnitude of differences between the patients of depression and control subjects in different samples (e.g., serum, plasma, RBCs) in acute episodes (AEs). Considering the effects of the treatment setting, we also quantified the changes after antidepressant therapy. Because no differences in oxidative stress markers between serum and plasma were found in our analysis, both materials were referred as “serum”. | 2 |
| **METHODS** | | |  |
| Protocol and registration | 5 |  |  |
| Eligibility criteria | 6 | Studies were included in our analyses if they met the following criteria: 1) cross-sectional studies of oxidative stress markers in serum, plasma, or RBC in human subjects; 2) studies that assessed oxidative stress markers in patients of depression in AEs at baseline and again after antidepressant therapy; 3) inclusion of a depression group as diagnosed by standard recognised criteria or screened with a standardised instrument; or 4) studies that provided subject numbers, means and standard deviations for oxidative stress markers. | 3 |
| Information sources | 7 | We searched Medline, Embase, PsychINFO, Sciencedirect, CBMDisc, CNKI and VIP from 1990 to May 2015. We hand-searched the references and selected the relevant articles for inclusion. | 3 |
| Search | 8 | We searched using following key words:(depression OR major depression OR unipolar depression OR major depressive disorder) AND (oxidative stress OR antioxidant OR antioxidant enzyme OR total antioxidant capacity OR total antioxidant potential OR free radical OR superoxide dismutase OR glutathione peroxidase OR catalase OR paraoxonase OR glutathione reductase OR vitamin C OR vitamin E OR albumin OR uric acid OR high-density lipoprotein cholesterol OR zinc OR nitric oxide OR nitrite OR peroxide OR malondialdehyde OR 8-F2-isoprostane OR oxidative neoepitope). |  |
| Study selection | 9 | We reviewed the titles and abstracts to select potentially relevant papers. Following this screening process, we reviewed the abstract of the papers. If there was doubt about the suitability of the paper based on the abstract alone, the full text was reviewed. | 3 |
| Data collection process | 10 | Data were extracted by two independent raters (TL and SZ) with disagreements settled by consensus and discussion. | 4 |
| Data items | 11 | Information was extracted in a systematic fashion as follows: 1) population characteristics; 2) sample type; 3) data for mean (SD); 4) diagnostic strategy; 5) treatment setting and 6) confounding factors. | 4 |
| Risk of bias in individual studies | 12 | The quality was assessed independently by two reviewers by using the Newcastle-Ottawa quality assessment scale (case control studies or cohort studies): 1) the selection; 2) the comparability; 3) the exposure or outcome. We identify ‘high’ quality choices with a ‘star’. A study can be awarded a maximum of one star for each numbered item within the selection and exposure categories. A maximum of two stars can be given for comparability or outcome. The studies with ≤4 stars were considered as low quality and were excluded | 4 |
| Summary measures | 13 | All statistical analyses were performed using standardized mean difference (SMD) methodology in Stata12.0 software. Pooled effect sizes were calculated for oxidative stress markers according to Hedges and Olgin for fixed effects models and DerSimonian and Laird for random effects models. A random effect model was chosen because of the diversity of methods, patients, clinical status, treatment, and so on. All comparisons were two-tailed, and 95% confidence intervals (CI) are described where applicable. (difference in means) | 4 |
| Synthesis of results | 14 | Pooled effect sizes were calculated for oxidative stress markers, including total antioxidant capacity (TAC), some antioxidant enzymes (SOD, GPX, CAT, PON and GR), antioxidants (albumin, uric acid, HDL-C, zinc, vitamin C and E), free radical (NO, nitric oxide, peroxide) and oxidative damage products (MDA, 8-*iso*-PGF_2α_, PCC) due to limited number of studies considering other oxidative stress markers. | 3 |

Page 1 of 2

| **Section/topic** | **#** | **Checklist item** | **Reported on page #** |
| --- | --- | --- | --- |
| Risk of bias across studies | 15 | Potential publication bias was assessed by using Egger’s test. Between-study heterogeneity (I^2^) was assessed as previously described by Glasziou and Sanders. | 4 |
| Additional analyses | 16 | Sensitivity analysis was performed by removing each study in the meta-analysis at one time to detect its influence on pooled effect sizes | 4 |
| **RESULTS** | | |  |
| Study selection | 17 | The search identified 7443 potentially relevant articles. After we removed duplicates, 6038 articles were remained. On the initial screening, 5882 were excluded based on titles and abstracts. Full-text evaluation was conducted for the remaining 156 articles, and 25 articles were excluded for not fulfilling inclusion criteria. 131 articles were remained. | 4 |
| Study characteristics | 18 | See table 1 |  |
| Risk of bias within studies | 19 | Though we systematically search, 131 articles were kept. However the quality of four papers was low, ranging from 3 to 4 stars in total. Eventually, 127 articles including 272 studies were included in our analyses | 4 |
| Results of individual studies | 20 |  |  |
| Synthesis of results | 21 | The serum TAC was decreased in AEs versus control subjects, but didn’t increase after a longitudinal anti-depressive treatment. Serum PON activity was decreased in AEs, whereas other serum enzymatic antioxidant activities activity didn’t differ in other analyses. There were no significant differences in RBC enzymatic antioxidant activities in any analyses. Serum uric acid, albumin, HDL-C and zinc levels were decreased in AEs versus control subjects, whereas serum uric acid and albumin levels were increased after a longitudinal anti-depressive treatment. Serum vitamin C levels were increased after anti-depressive treatment. There were no significant differences in serum vitamin C and vitamin E levels in other analyses. It were increased in serum peroxide levels in AE versus control group. Serum nitrite levels were decreased after a longitudinal anti-depressive treatment, whereas serum nitrite levels didn’t differ in any other analyses. There were not differences in serum NO levels in any performed analyses. There were significantly increased levels of RBC MDA in AE, serum MDA in AE, serum 8-*iso*-PGF_2α_ in AE, whereas RBC MDA and serum MDA levels were decreased after a longitudinal anti-depressive treatment. Serum PCC levels didn’t differ between patients and controls in any analyses which could be performed. | 5-7 |
| Risk of bias across studies | 22 | Publication bias assessed with Egger’s test was significant for analyses for GPX in CBAT, CAT in CBAT and GR in AEs, vitamin C in CBAT, vitamin E in AEs, uric acid in AEs, albumin in AEs and CBAT, and HDL-C in AEs, peroxide levels in AEs, RBC and serum MDA in AEs, but not other analyses which could be performed. Publication bias for other oxidative stress marker analyses couldn’t be performed because of limited studies.  The heterogeneity was high in ES estimates for serum TAC in AEs and CBAT; RBC SOD, GPX, GR in AEs and CBAT, RBC CAT in AEs; serum SOD, GPX, CAT, PON in AEs and CBAT; serum uric acid, albumin, HDL-C and zinc, vitamin C and vitamin E in AEs, serum uric acid, albumin, zinc and vitamin E in CBAT; nitrite, NO and peroxide levels in AEs; RBC MDA, serum MDA, 8-iso-PGF2α, PCC in AEs, serum MDA, 8-iso-PGF2α, PCC in CBAT but not other analyses. | 5-7 |
| Additional analysis | 23 | In sensitivity analyses, the heterogeneity was no longer significant, serum TAC in CBAT remained unchanged by removal of two studies, RBC SOD activity changed to be decreased after anti-depressive treatment after removal of two studies, and RBC GPX in AE changed to be increased versus control subjects after removal of two studies, and RBC CAT in AE and drug-naive AE levels still didn’t differ between groups after removal of one study, serum SOD activity changed to be decreased after a longitudinal anti-depressive treatment after removal of one study, serum vitamin C level remained increased after anti-depressive treatment after removal of two study, serum albumin level in drug-naive AE remained decreased after removal of one study, serum peroxide level in AE remained increased after removal of one study, RBC MDA level in AE and serum MDA level in drug-naive AE remained increased, serum MDA level remained decreased after anti-depressive treatment versus control group, serum PCC level in AE still didn’t differ between groups after removal of one, two, two and one studies respectively. There was also significant heterogeneity for some another analyses after removal of each single study and all combinations of two studies. Sensitivity analyses were not possible for some another parameters. | 5-7 |
| **DISCUSSION** | | |  |
| Summary of evidence | 24 | So the present findings support that the oxidative stress dysregulation in depressed patients might mainly due to that lower antioxidants intake, leading to the impaired antioxidant defense (lower TAC). Impaired antioxidant defense can’t scavenge free radicals in time, leading to free radicals increased gradually, and higher activities of enzymatic antioxidants might be a compensatory mechanism to excessive production of free radicals, such as RBC SOD and GPX. Excessive free radical levels caused damage to main parts of cellular macromolecules, such as fatty acids, protein, DNA, RNA and mitochondria, and the longitudinal anti-depressive treatment can reverse these abnormal oxidative stress parameters. | 7 |
| Limitations | 25 | Several limitations are few studies, public bias and between-study heterogeneity in these meta-analyses, results for many oxidative stress markers should be interpreted with caution | 7 |
| Conclusions | 26 | The results of the present meta-analysis reveal that oxidative stress is disturbed in individuals with depression. The findings suggest the need to further investigate in the potential roles of oxidative stress biomarkers in the pathophysiology of depression, the potential utility of antioxidant biomarkers and its clinical application, and potential benefits of antioxidants supplementation in depressed patients. | 9 |
| **FUNDING** | | |  |
| Funding | 27 | YBJ was supported by the Fundamental Research Funds for the Guangdong Provincial Department of Education (ID: 2013KJCX0025), and MOE (Ministry of Education in China) of Humanities and Social Sciences Project (ID: 13YJA190008). TL was supported by the funds from“Challenge Cup” National Science and Technology College of Extracurricular Academic Competition in China. |  |

*From:*  Moher D, Liberati A, Tetzlaff J, Altman DG, The PRISMA Group (2009). Preferred Reporting Items for Systematic Reviews and Meta-Analyses: The PRISMA Statement. PLoS Med 6(6): e1000097. doi:10.1371/journal.pmed1000097

For more information, visit: **www.prisma-statement.org**.

Page 2 of 2
